# Evaluating hybridization capture with RAD probes as a tool for museum genomics with historical bird specimens

**DOI:** 10.1002/ece3.3065

**Published:** 2017-05-23

**Authors:** Ethan B. Linck, Zachary R. Hanna, Anna Sellas, John P. Dumbacher

**Affiliations:** ^1^ Department of BiologyBurke Museum of Natural History & CultureUniversity of WashingtonSeattleWAUSA; ^2^ Museum of Vertebrate ZoologyUniversity of California, BerkeleyBerkeleyCAUSA; ^3^ Department of Integrative BiologyUniversity of California, BerkeleyBerkeleyCAUSA; ^4^ Ornithology & MammologyCalifornia Academy of SciencesSan FranciscoCAUSA; ^5^ Center for Comparative GenomicsCalifornia Academy of SciencesSan FranciscoCAUSA

**Keywords:** ancient DNA, hyRAD, museum genomics, RADseq, sequence capture

## Abstract

Laboratory techniques for high‐throughput sequencing have enhanced our ability to generate DNA sequence data from millions of natural history specimens collected prior to the molecular era, but remain poorly tested at shallower evolutionary time scales. Hybridization capture using restriction site‐associated DNA probes (hyRAD) is a recently developed method for population genomics with museum specimens. The hyRAD method employs fragments produced in a restriction site‐associated double digestion as the basis for probes that capture orthologous loci in samples of interest. While promising in that it does not require a reference genome, hyRAD has yet to be applied across study systems in independent laboratories. Here, we provide an independent assessment of the effectiveness of hyRAD on both fresh avian tissue and dried tissue from museum specimens up to 140 years old and investigate how variable quantities of input DNA affect sequencing, assembly, and population genetic inference. We present a modified bench protocol and bioinformatics pipeline, including three steps for detection and removal of microbial and mitochondrial DNA contaminants. We confirm that hyRAD is an effective tool for sampling thousands of orthologous SNPs from historic museum specimens to describe phylogeographic patterns. We find that modern DNA performs significantly better than historical DNA better during sequencing but that assembly performance is largely equivalent. We also find that the quantity of input DNA predicts %GC content of assembled contiguous sequences, suggesting PCR bias. We caution against sampling schemes that include taxonomic or geographic autocorrelation across modern and historic samples.

## 1 INTRODUCTION

Over the past three decades, novel laboratory techniques have enhanced our ability to generate DNA sequence data from millions of natural history specimens collected prior to the molecular era (Payne & Sorenson, 2002). The advent of ancient DNA methods has allowed researchers to obtain both nuclear and mitochondrial DNA (mtDNA) sequences from extinct taxa (Cooper et al., 1992; Fleischer et al., 2006), explore changes in genetic diversity and population genetic structure over time (Habel, Husemann, Finger, Danley, & Zachos, 2014; Weber, Stewart, Garza, & Lehman, 2000), incorporate threatened or difficult‐to‐collect taxa into population genetic or phylogenetic studies (Guschanski et al., 2013; Linck, Schaack, & Dumbacher, 2016), and take advantage of extant biological collections to boost sample size and inferential power (Linck, Schaack, & Dumbacher, 2016; Wójcik, Kawałko, Marková, Searle, & Kotlík, 2010). Now, high‐throughput sequencing has dramatically increased both the overall efficiency of data collection and the total amount of sequence data that it is possible to collect from museum specimens (Hofreiter et al., 2015; Rizzi, Lari, Gigli, De Bellis, & Caramelli, 2012) by overcoming scalability hurdles intrinsic to traditional Sanger sequencing methods (Soltis & Soltis, 1993; Wandeler, Hoeck, & Keller, 2007).

Although high‐throughput sequencing has already proved widely useful for incorporating museum specimens into phylogenomic studies (Besnard et al., 2015; Burbano et al., 2010; McCormack et al., 2012), its application for collecting genome‐wide markers at the population level has lagged behind its use for addressing questions at deeper evolutionary time scales due to limitations in the most commonly employed library preparation methods for reduced‐representation Illumina sequencing (Suchan et al., 2016). The limitations of historic museum samples include their high degree of fragmentation and low concentration of long DNA fragments, which reduces the amount of flanking sequence that can be captured using ultraconserved element probes (Faircloth et al., 2012) and lowers the likelihood that multiple restriction digest recognition sequences are retained in a given DNA fragment (Baird et al., 2008; Peterson, Weber, Kay, Fisher, & Hoekstra, 2012). Only in the past few years have library preparation protocols suitable for population genomics become available (Bi et al., 2013; Jones & Good, 2016; McCormack, Tsai, & Faircloth, 2016), but their recent proliferation has meant that few have yet to be applied to multiple study systems in independent laboratories (McCormack et al., 2016). As a result, our understanding of the efficacy and biases of different approaches to reduced‐representation genome sequencing from degraded DNA remains incomplete relative to either Sanger sequencing (Soltis & Soltis, 1993; Wandeler et al., 2007) or high‐coverage, single‐sample whole genome sequencing (Poinar et al., 2006).

One promising but under‐tested approach to museum genomics suitable for population‐level studies is hybridization capture of restriction site‐associated DNA (RAD) probes (hyRAD) (Suchan et al., 2016). Briefly summarized, the hyRAD method uses fragments produced by a double digest RAD (ddRAD) protocol (Peterson et al., 2012; Suchan et al., 2016) as the basis for biotinylated probes that capture orthologous loci in other samples, allowing them to be enriched and indexed for pooled Illumina sequencing. Although the method requires a high molecular weight DNA sample to produce the probe set, hyRAD offers advantages over other targeted capture methods in requiring no prior knowledge of the organism's genome, such as transcriptome data or pre‐existing sequences for probe design (Bi et al., 2013; McCormack et al., 2016). Additionally, because hyRAD relies on hybridization capture of orthologous regions across samples rather than retained restriction‐site recognition sequences, the method mitigates the concerns of allelic dropout due to polymorphisms at restriction sites with increasing phylogenetic distance intrinsic to other RAD‐based protocols (Gautier et al., 2013).

In their original paper, Suchan et al. (2016) validated their method by applying it to both fresh tissue and museum specimens of a butterfly (*Lycaena helle*) and grasshopper (*Oedaleus decorus*). They discussed the impact of library preparation, sample type, and bioinformatics pipeline on the number of SNPs produced. Here, we provide an independent assessment of the effectiveness of hyRAD using both fresh avian tissues and dried tissue taken from museum specimens up to 140 years old. We present a modified version of the hyRAD protocol aimed at increasing efficiency and minimizing reagent use and employ a custom bioinformatics pipeline with steps for detecting and removing microbial contamination in raw reads, contiguous sequences, and SNPs. We utilize hyRAD data to describe phylogeographic patterns in a New Guinea forest kingfisher (*Syma torotoro*) and we expand the available description of hyRAD's performance by investigating how variable input DNA affects sequencing, assembly, and population genetic inferences.

## 2 METHODS

### 2.1 Study species, sampling, and DNA extraction

A major promise of museum genomics is the ability to conduct population‐level studies in regions that are too logistically difficult to be amenable to broad modern sampling programs. The island of New Guinea is an apt example of this scenario, with poorly known biodiversity, large historical collections, rugged terrain, and ongoing political instability (Mack & Dumbacher, 2007; Pratt & Beehler, 2014). Phylogeographic research in New Guinea has been limited (Deiner, Lemmon, Mack, Fleischer, & Dumbacher, 2011; Dumbacher & Fleischer, 2001), especially in the species inhabiting the island's ring of lowland tropical rainforest. To evaluate the efficacy of for use in a broader study of the phylogeography of lowland new Guinea, we sampled 21 individuals of forest interior resident *S. torotoro* (Yellow‐billed Kingfisher), representing five named subspecies and the breadth of the species’ range on the island of New Guinea (Table 1). For seven individuals, we extracted whole‐genomic DNA from fresh tissue using a DNeasy tissue extraction kit (Qiagen, Valencia, CA, USA) following the manufacturer's protocol. For the remaining 14 individuals, we extracted DNA from the toe‐pads of museum study skins in a dedicated ancient DNA laboratory at the California Academy of Sciences using a phenol–chloroform and centrifugal dialysis method described elsewhere (Dumbacher & Fleischer, 2001). No modern DNA or post‐PCR products are handled in this laboratory, which is located on a separate floor from the main genetics facility.

**Table 1:** Sampling information

Specimen	Subspecies	Sample type	Locality	Date collected	Age of sample (years)	Total number of reads	Number of cleaned reads	% Duplicate reads	Average coverage (X)	Specificity	Fold enrichment	Sensitivity	Loci	Average locus length (nt)	Number of contigs over 1KB	Initial concentration (ng/μl)	GC content of on‐target contigs
KU:Birds:5215	*pseuestes*	Tissue	Ivimka Camp, Gulf Province, Papua New Guinea	2003	13	18,713,496	2,915,214	68.1	12.74	16.87	16.58	84.93	22,568	518	489	71.6	44.22
KU:Birds:5464	*pseuestes*	Tissue	Sapoa Camp, Gulf Province, Papua New Guinea	2003	13	17,246,396	2,512,928	68.2	11.2	16.65	16.36	79.43	19,880	499	340	51.8	44.69
KU:Birds:6927	*meeki*	Tissue	Mt. Suckling, Oro Province, Papua New Guinea	2011	5	25,892,086	3,103,912	73.2	14.46	17.37	17.07	85.02	23,162	510	494	37	44.53
KU:Birds:7131	*pseuestes*	Tissue	Dark End Camp, Gulf Province, Papua New Guinea	2002	14	6,819,041	1,154,541	60.4	5.87	17.29	16.99	65.44	12,725	434	157	31.8	45.01
CAS:ORN:626	*meeki*	Blood	Varirata National Park, Central Province, Papua New Guinea	2011	5	15,548,547	1,945,868	70.2	9.38	17.06	16.77	77.25	18,519	488	262	43	44.62
KU:Birds:9145	*torotoro*	Tissue	Gahom Camp, East Sepik Province, PNG	2003	13	18,049,058	2,309,716	70.2	10.75	16.91	16.62	80.15	20,057	452	312	10.1	44.35
AMNH:Birds:637445	*torotoro*	Toe pad	Wasior, West Papua Province, Indonesia	1928	88	13,112,780	48,257	78.2	2.59	11.16	10.97	33.52	849	449	57	3.66	46.03
AMNH:Birds:329542	*ochracea*	Toe pad	Sewa Bay, Normanby Island, Papua New Guinea	1934	82	15,875,059	40,402	82.2	1.8	8.9	8.75	29.19	388	465	24	3.84	47.62
AMNH:Birds:637464	*tentelare*	Toe pad	Wannambai, Maluku Province, Indonesia	1896	120	21,058,924	139,248	59.9	9.2	11.07	10.88	50.24	3,197	449	154	10.2	44.98
AMNH:Birds:637450	*torotoro*	Toe pad	Humbolt Bay, Papua Province, Indonesia	1928	88	3,692,636	16,359	75.1	0.89	12.06	11.85	14.62	153	543	15	0.714	48.74
AMNH:Birds:637429	*torotoro*	Toe pad	Misool Island, West Papua Province, Indonesia	1900	116	13,531,142	35,925	79.8	2.13	9.46	9.30	30.09	412	448	26	2.54	46.44
AMNH:Birds:300723	*torotoro*	Toe pad	Waigeu Island, West Papua Province, Indonesia	1900	116	14,143,466	34,596	82.2	1.91	10.03	9.86	27.78	508	401	21	0.846	46.88
AMNH:Birds:637446	*torotoro*	Toe pad	Kepaur, West Papua Province, Indonesia	1897	119	20,487,169	36,627	84.1	2.15	8.02	7.88	23.19	177	600	22	1.07	48.18
NHMUK:ZOO:1911.12.20.823	*pseuestes*	Toe pad	Mimika River, Papua Province, Indonesia	1913	103	2,875,704	19,115	50.9	1.55	10.85	10.66	20.44	239	611	40	1.49	47.28
NHMUK:ZOO:1911.12.20.822	*pseuestes*	Toe pad	Satakwa River, Papua Province, Indonesia	1911	105	5,464,605	46,548	59.8	2.08	9.44	9.28	24.39	296	579	36	0.584	48.32
AMNH:Birds:437798	*torotoro*	Toe pad	Amberbaki, West Papua Province, Indonesia	1877	139	1,579,610	4,957	73.4	0.39	9.1	8.94	4.58	52	582	4	1.84	48.88
AMNH:Birds:637441	*torotoro*	Toe pad	Mt. Mori, West Papua Province, Indonesia	1899	117	6,803,386	19,623	76.9	1.3	9.5	9.34	21.38	157	512	11	7.36	47.89
AMNH:Birds:293741	*torotoro*	Toe pad	Ifaar, Papua Province, Indonesia	1928	88	27,602,106	49,786	91.1	2.14	12.48	12.27	23.84	493	447	28	0.124	50.41
AMNH:Birds:293715	*torotoro*	Toe pad	Kepaur, West Papua Province, Indonesia	1928	88	30,751,249	56,972	90.5	2.15	11.15	10.96	24.2	438	504	30	0.15	50.95

### 2.2 Library preparation, hybridization capture experiments, and sequencing

We prepared samples for reduced‐representation whole genome sequencing using a modified version (Hanna & Sellas, 2016) of Suchan et al.'s (2016) hyRAD method aimed at increasing efficiency of reactions and reducing reagent use. We present this protocol in a detailed bench‐ready version online (https://github.com/calacademy-research/hyRADccg) and summarize it below.

To produce biotinylated probes, we performed a double restriction digest with enzymes MluCl and SphI (New England Biolabs) on 400 ng of high molecular weight DNA extracted from fresh tissue of a single *S. t. ochracea* individual. After ligation of adapters to fragments, we size‐selected the resulting fragments on a Pippin Prep (Sage Science, Beverly, MA, USA) with a target peak at 270 bp and “tight” size selection range. We ran 16 cycles of real‐time polymerase chain reaction (RT‐PCR) and purified products by gel excision and a Zymoclean Gel Recovery Kit (Zymo Research). We preserved one aliquot of this product for sequencing while performing an additional MluCl/SphI double digest to de‐adapterize a second aliquot. We labeled this deadapterized aliquot with biotin‐14‐dATP, using a BioNick DNA Labeling System (Thermofisher Scientific).

To produce whole genome libraries, we sheared high molecular weight DNA from modern tissue samples to ~400 bp using a M‐220 Focused‐ultrasonicator (Covaris). DNA from museum specimen toepads was already fragmented as a product of natural degradation associated with the age of the samples and was therefore left untreated. For both modern and historic samples, we used a Kapa Hyper Prep Kit (Kapa Biosystems) to prepare dual‐indexed libraries. We amplified libraries using 5–13 cycles of RT‐PCR. After quantifying DNA content in each sample, we made standardized dilutions of each sample and combined equal amounts of these dilutions to create one pool of modern DNA samples (*n* = 6) and two pools of ancient DNA samples (*n* = 7 each). We used a 1–1.5× ratio of AmPure XP beads to remove small DNA fragments throughout the protocol and assessed DNA quantity and quality with a Qubit 2.0 fluorometer and an Agilent 2100 Bioanalyzer between all major steps.

To perform hybridization capture reactions, we incubated each pool of samples with 250 ng of biotinylated probe for 72 hr at 55°C in a solution containing 20× saline‐sodium citrate (SSC), 50× Denhardt's Solution, 0.5 mol/L EDTA, 10% sodium dodecyl sulfate (SDS), and a blockers mix containing Chicken Hybloc (0.5 μg/μl), IDT's xGen Universal Blocking Oligo ‐TSHT‐ i5 (0.05 nmol/μl), and IDT's xGen Universal Blocking Oligo ‐TSHT‐ i7 (0.05 nmol/μl). Following hybridization, we prepared 50 μl Dynabeads MyOne Streptavidin C1 beads for use by washing three times with 1× binding buffer containing 2 mol/L NaCl, 10 mmol/L Tris–HCl (pH 7.5), 0.5% Polysorbate 20 (Tween‐20), and 1 mmol/L EDTA, and final resuspension in 70 μl 2× binding buffer. We then bound the probes to the beads by mixing and incubating at room temperature for 30 min. After performing three 500 μl washes of the bead‐probe mixture using a prewarmed buffer containing 10% SDS with 0.1× SSC, we concentrated our final pooled libraries in 30 μl 10 mm Tris–HCl, 0.05% Tween‐20 (pH 8–8.5). We next amplified these libraries using RT‐PCR for 9–2 cycles, cleaned using 1.2× Ampure XP beads, and quantified using Qubit. We sent a single final pool with equimolar amounts of all three hybridized pools to University of California Bekeley's QB3 Vincent J. Coates Genomics Sequencing Laboratory (hereafter called “QB3”) for sequencing with 100‐bp paired‐end sequence reads on a single lane of an Illumina HiSeq 4000.

### 2.3 Sequence read quality control, assembly, and alignment

**Figure 1:**
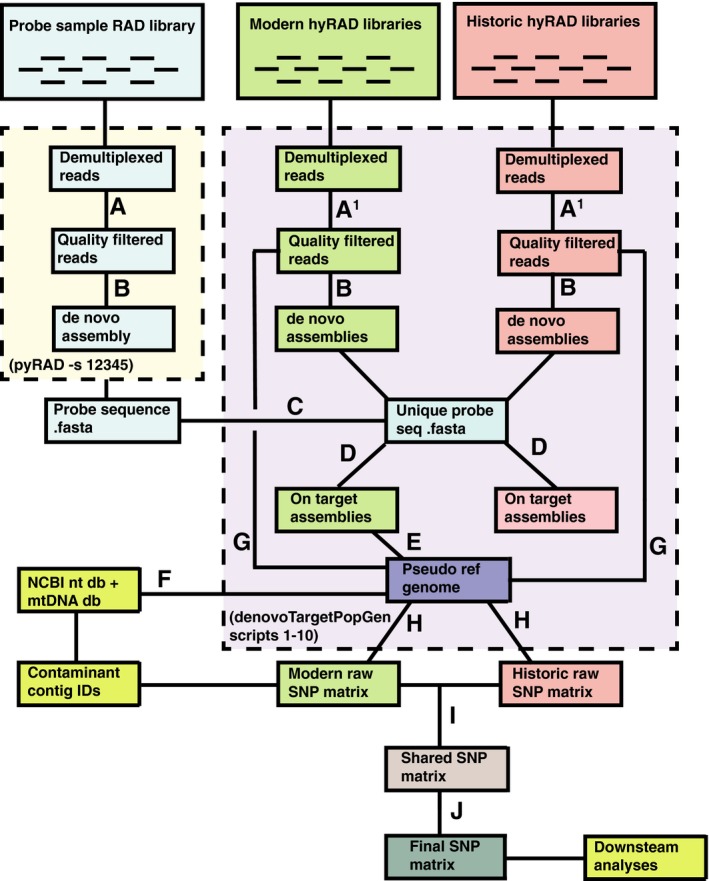
Bioinformatics pipeline for *S. torotoro* hyRAD data. We demultiplexed 100‐bp paired‐end reads from three genomic libraries and filtered for adapter contamination/quality scores (A), or adapter contamination/quality scores and *E. coli* contamination (A1). Reads were clustered (as consensus fasta files) (B), and repeat regions removed from probes (C). After determining which assembled clusters were orthologous with probe regions (D), we merged flanking regions from on‐target loci in modern samples with the repeat‐free probe sequence to create a pseudo‐reference genome (E). To identify which contigs represented contamination in the original probe sample library from exogenous microbes or mitochondrial DNA, we BLAST searched against both the NCBI nt database and a full mitochondrial genome from *S. torotoro* relative *Halcyon sanctus* (F). We aligned quality filtered reads to this pseudo reference (G), called SNPs to produce a raw.vcf file for historic and modern DNA libraries separately (H). After filtering SNPs for origin in contaminant contigs and then restricting our matrix to sites present in both sample types (I), we filtered SNPs by read depth, quality scores, probability of being variable sites, and minor allele frequencies (J) prior to downstream analyses

To clean and quality filter reads, assemble reads into contigs, align sequences across samples, and map reads to merged alignments for SNP discovery, we used a custom pipeline combining in‐house R scripts as well as pre‐existing genomics tools and wrapper scripts from QB3's two de novo targeted capture bioinformatics pipelines (https://github.com/CGRL-QB3-UCBerkeley; “denovoTargetCapturePopGen” and “denovoTargetCapturePhylogenomics”). We present our full pipeline online as both a tutorial and a list of shell commands (https://github.com/elinck/hyRAD/) (Figure 1).

We first processed reads from our probe library with pyRAD version 2.17 (Eaton, 2014) to create a pseudo‐reference genome to use as the basis for aligning sequences from samples in our hybridization capture reactions. After quality‐filtering reads and trimming adapter contamination, pyRAD used the vsearch algorithm (Rognes, Flouri, Nichols, Quince, & Mahé, 2016) to cluster reads into loci within samples, cluster loci into stacks between samples, and aligned putatively orthologous loci using MUSCLE version 3.8 (Edgar, 2004). We implemented strict adapter filtering, retained reads longer than 70 bp after trimming, set a minimum sequence identity threshold of 97% for clustering, and kept four sites per cluster with a Phred Quality Score <20. (This strict identity threshold was selected to generate an estimate of the total number of fragments in our probe library.) We removed repetitive genomic regions and paralogs with the NCBI BLAST+ version 2.4 tool BLASTn (Boratyn et al., 2013) by aligning the output file of assembled clusters against itself and retained only cluster sequences that aligned uniquely to themselves using an *e*‐value of 0.00001.

To remove reads that failed to pass Illumina quality control filters, trim reads for quality and adapter contamination, merge overlapping reads, remove PCR duplicates, and remove endogenous *E. coli* contamination, we used QB3's denovoTargetCapturePopGen “2‐ScrubReads” wrapper around the Trimmomatic version 0.36 (Bolger, Lohse, & Usadel, 2014), Bowtie 2 (Langmead, 2010), Cutadapt (Martin, 2011), Cope (Liu et al., 2012), FastQC (http://www.bioinformatics.babraham.ac.uk/projects/fastqc/), and FLASh (Magoc & Salzberg, 2011) tools. We assembled cleaned and filtered reads for each sample using QB3's denovoTargetCapturePhylogenomics wrapper script “2‐GenerateAssembliesPhylo” around the SPAdes version 3.8.1 genome assembler (Bankevich et al., 2012), which automatically selects a k*‐*mer value based on read length and dataset type. To determine which contigs from our capture libraries were orthologous with probe regions, we used the denovoTargetCapturePopGen wrapper “5‐FindingTargets” around the BLAST+ (Boratyn et al., 2013) and cd‐hit‐est (Fu, Niu, Zhu, Wu, & Li, 2012) tools. Analyzing samples from modern and historical DNA separately, we used a clustering identity threshold of 95% and permitted 100 bp of sequencing flanking the core probe region. After determining matches, we collapsed overlapping, orthologous contigs from all modern samples with the probe library to generate an extended pseudo‐reference genome to which we aligned cleaned reads using QB3's denovoTargetCapturePopGen wrapper “7‐Alignment” around the Novoalign version 3.04.06 tool (http://www.novocraft.com/products/novoalign/). We ran Novoalign with an average library insert size of 235 and a maximum alignment score of 90.

### 2.4 SNP discovery

Traditional SNP calling algorithms based on allele counting and quality scores are characterized by high degrees of uncertainty with low‐coverage sequence data (Korneliussen, Albrechtsen, & Nielsen, 2014). We incorporated uncertainty into genotype estimation by calling SNPs and estimating allele frequencies using an empirical Bayesian framework implemented in the software ANGSD version 0.913 (http://www.popgen.dk/angsd/index.php/ANGSD). ANGSD uses the likelihood of all 10 possible genotypic configurations for each site passing quality filters in all individuals to estimate a site frequency spectrum, which is then used as a prior to estimate the posterior probabilities for all possible allele frequencies at each site in each sample. Using these estimates, we called SNPs with a 95% probability of being variable and a minimum minor allele frequency of 5%.

### 2.5 Contamination control and data filtering

In order to identify if any contigs in our assemblies represented off‐target mtDNA captures, we performed a BLAST+ (Boratyn et al., 2013) nucleotide search with each of our assemblies as a query against a database of the full mitochondrial genome of *S. torotoro* relative *Halcyon santcus*. We then removed all matching contigs from each sample's assembly fasta with in‐house R scripts (“excerptcontigIDs.R” and “cutcontigsbatch.R”) and used these mtDNA‐free sequences for all subsequent assembly performance calculations. To prepare our sequence alignment in.sam/.bam format for SNP calling, we followed Bi et al. (2013) in hierarchically filtering out individuals, contigs, and sites that appeared to be quality outliers and implemented additional steps for regions derived from microbial contamination or mtDNA. We determined no individuals had abnormal coverage (defined as <1/3 or >3× the average coverage across all individuals) and we created merged, sorted BAM files and generated raw variant call format files (.vcf) with samtools version 1.3 (Li et al., 2009) and bcftools version 1.3.1 (Narasimhan et al., 2016), processing modern and historical DNA samples separately.

Because our SNP matrix was derived independently from our assemblies, we performed a second step of contamination filtering by removing SNPs originating from read alignments to regions of exogenous microbial DNA and/or mtDNA present in the original probe sample RAD library. We used our full pseudo‐reference genome as the query in a search of the entire BLAST+ (Boratyn et al., 2013) nucleotide database and our *H. sanctus* mtDNA BLAST+ database. We then used Henderson and Hanna's (2016) “GItaxidIsVert.py” script to identify sequences that were potentially microbial in origin and performed a second BLAST+ search with this subset to further select only the subset of contigs that had their best or only alignment with nonvertebrate reference genomes. To exclude such sequences as well as those aligning with mtDNA sequence, we used the vcftools version 0.1.11 “–not‐chr” flag and removed indels in the same step with the “–remove‐indels” flag.

We estimated independent empirical gene coverage and site depth distributions using QB3's denovoTargetCapturePopGen “9‐preFiltering” script and used these distributions as input to the QB3 “10‐SNPcleaner” script. Run separately for modern and historical samples, this script removed all sites with coverage below 6×, sites missing in more than half of our samples, and sites with variant identity biases associated with quality score, mapping quality, or distance of alleles from the ends of reads. Because hydrolytic deamination of cytosine (C) to uracil (U) residues is the most common form postmortem nucleotide damage present in historic museum specimens, which may result in misincorporation of thymines (Ts) instead of uracil during PCR amplification and bias population genetic inference, this script also eliminated all C to T and G to A SNPs (Axelsson, Willerslev, Gilbert, & Nielsen, 2008; Briggs et al., 2007; Hofreiter, Jaenicke, Serre, von Haeseler, & Pääbo, 2001). Finally, we used the BEDtools version 2.26.0 “intersect” function (Quinlan & Hall, 2010) to retain only the sites that passed all filters for both historic and modern specimens.

### 2.6 Statistical analyses

To assess the differences in sequencing and assembly performance between modern and historical samples, we implemented Wright's two‐sample *t* tests in the R (R Core Team 2016). We evaluated differences between groups in the mean percentage of duplicate reads, the mean number of on‐target contigs, the mean length of on‐target contigs, and the percentage of sequenced reads successfully mapping to our pseudo‐reference genome. Because some commonly used polymerases bias against amplification of targeted DNA in favor of the GC‐rich microbial contamination common to extracts from museum specimens (Dabney & Meyer, 2012), we assessed differences in GC content present in assembled on‐target contigs. In order to determine whether sample age, initial sample DNA concentration, or sequencing effort were significant predictors of %GC content among historical samples and mean number or length of on‐target contigs, we used simple linear regression. We used stepwise model selection with corrected Akaike information criterion scores to determine best‐fit models and did not include interaction terms to avoid over‐parameterization given our small sample size.

### 2.7 Population genetic clustering and discriminant analysis of principal components

Although accurate estimation of population genetic structure in *S. torotoro* was not the primary goal of our study, we were nonetheless interested in assessing hyRAD's ability to produce biologically meaningful results by testing whether our data reflected the signature of phylogeographic processes such as isolation by distance (IBD) and vicariance, rather than the signature of DNA degradation, contamination, or other artifacts of library preparation and sequencing. We implemented *k*‐means clustering and discriminant analysis of principal components (DAPC) in the R package adegenet (Jombart, 2008), using 100% complete data matrix (1,690 SNPs) to avoid biasing inferences with nonrandom patterns of missing data. We retained all principal component (PC) axes for *k*‐means clustering and inspected both population assignments and change in Bayesian information criterion (BIC) scores across multiple values of *K* to select an optimal partitioning scheme. To maximize among‐population variation and calculate ancestral population membership probabilities for each sample, we performed DAPC on the first six PCs using two discriminant axes. We then chose to retain these six PCs to optimize the *a‐*score value for our data, which is the difference between the proportion of successful reassignment of the analysis and values obtained using random groups. However, because the change in BIC scores failed to clearly indicate any “true” value of *K*, we repeated our analysis with clustering assignments for *K* values of 1–8. To explore correlations between our three retained PCs and variables expected to differ between modern and historic samples (specificity, sensitivity, fold enrichment, age, initial concentration), we again performed simple linear regressions. Finally, to test for patterns of IBD across our samples, we performed a Mantel test among all individuals based on 999 simulated replicates using the R package ade4 (Thioulouse & Dray, 2007).

## 3 RESULTS

### 3.1 Hybridization capture experiments and sequencing

We obtained a total of 397 million sequence reads for the probe and hybridization capture libraries, successfully demultiplexing 20/21 samples, with one sample failing due to bar code error. The total reads per sample ranged from 1.6 million to 30.7 million, and the average number of reads per sample did not vary significantly between modern and historical samples (*t = *−0.946, *df* = 14.6, *p = *.359). Of the original 397 million reads, 19.8% passed initial Illumina quality filters, contamination checks, adapter trimming, and removal of PCR duplicates (Table 1). The resulting number of cleaned reads per sample ranged from approximately 16,000 to 3.1 million, with an average count of 766,662, and significantly fewer reads for historic samples (*t = *−3.185, *df *= 13.088, *p = *.007). Most reads lost to quality control were PCR duplicates, with a range of 50.9%–91.1% duplicate reads per sample. 2,455 reads were removed as *E. coli* contamination from 12 of 20 individuals (range 1–2,318 reads per individual) (Table 2). The average depth of read coverage per sample, calculated as the read depth per base averaged across the length of the pseudo‐reference genome, ranged from 7.6 to 26.4, and was significantly lower in historic samples (*t = *−3.754, *df *= 12.632, *p =* .002).

**Table 2:** Results of microbial and mitochondrial contamination removal at three distinct steps. Raw reads were filtered for *E. coli* contamination; assemblied contigs were filtered for mitochondrial DNA; SNP matrices were filtered for both microbial DNA and mitochondrial DNA

Contamination filtering step	Sample type	Total count	Count removed	Percent removed
Raw reads	Modern	13,942,179	4	<0.001
	Historic	548,415	2,451	0.44
Assembled contigs	Modern	296,828	8	<0.001
	Historic	36,109	53	0.15
SNP matrix	Modern	6,915,902	6,620	<0.001
	Historic	749,091	3,945	0.53

### 3.2 Assembly and alignment results

**Figure 2:**
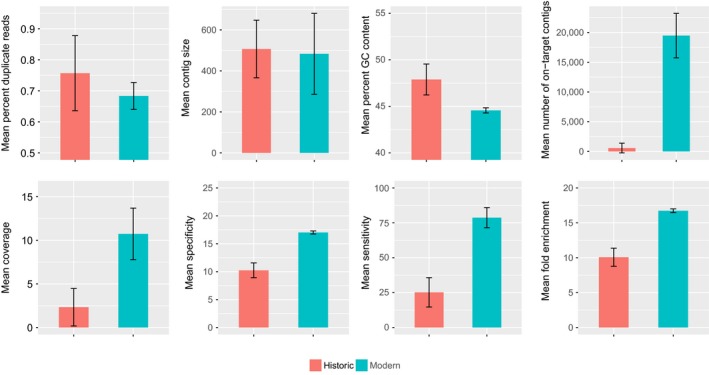
Differences in sequencing and assembly performance between historical and modern DNA extractions. We observed significantly higher specificity (*t* = −17.711, *df *= 14.015, *p *< .001), sensitivity (*t* = –12.928, *df *= 14.014, *p *< .001), fold enrichment (*t* = −17.711, *df* = 14.015, *p *< .001), and average coverage (*t* = −6.248, *df *= 7.555, *p *< .001) in modern samples. We recovered a significantly higher total number of on‐target loci in modern samples (*t* = −12.239, *df *= 5.221, *p *< .001), but significantly higher mean percent GC content in historic samples (*t* = 6.997, *df* = 13.368, *p *< .001). We observed no significant differences between modern and historic samples for mean contig size or mean percentage of duplicate reads

**Figure 3:**
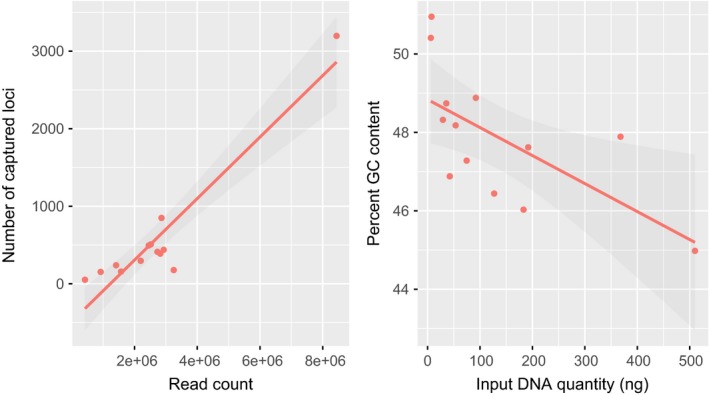
Among historic samples, the number of trimmed reads was a significant predictor of the number of captured loci (*R* ^2^ = .872, *p *< .001) and the initial sample DNA input quantity was a significant predictor of percent GC content in on‐target assembled contigs (*R* ^2^ = .370, *p *= .016)

Assembly of our probe library in pyRAD resulted in a total of 554,048 contigs and 61.9 million nucleotides (nt), which was reduced to 160,014 unique contigs and 16.1 million nt after excluding repetitive regions. We captured orthologous loci from all successfully sequenced samples, and after merging the probe library with flanking regions from assemblies of other modern tissue samples our extended pseudo‐reference genome contained 29,297 loci. The number of contigs per sample that was orthologous to our probe library ranged from 55 to 23,155 and was significantly higher in modern samples (Table 1; Figure 2). Across all samples, we discarded 55%–92% of the total number of assembled clusters as off‐target loci, losing significantly more from historic samples (*t* = 6.6035, *df* = 16.889, *p *< .001). We removed an additional eight contigs from the modern samples and 53 contigs from the historic samples due to their mitochondrial origin (Table 2). Mean contig size ranged from 401 to 611 nt and did not differ significantly between modern and historic samples. However, the historic samples had significantly fewer contigs exceeding 1 knt in length (*t = *–3.181, *df *= 5.004, *p = *.025). The percentage of reads passing quality filters that successfully mapped to the pseudo‐reference genome (also known as specificity) ranged from 51.8% to 57.7% and was significantly higher on average for modern samples (Figure 2). Additionally, %GC content was significantly higher in historic than modern samples (Figure 2). Among historic samples, the number of cleaned reads was a significant predictor of the number of captured loci and sample input DNA quantity was a significant predictor of %GC content in on‐target assembled contigs (Figure 3).

### 3.3 SNP discovery and filtering

**Figure 4:**
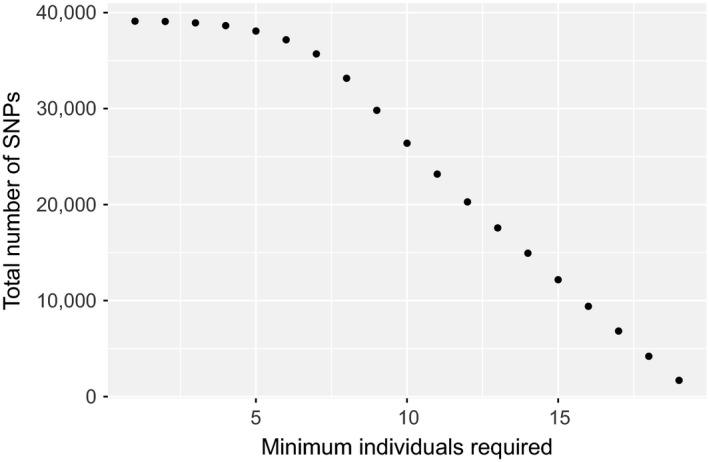
This displays the relationship between the cutoff for the minimum number of genotyped individuals required for a given SNP to be included in the data matrix and the total number of SNPs included in the resulting matrix

We identified two contigs of mitochondrial origin and eight contigs of potential nonvertebrate origin in our pseudo‐reference genome and excluded all sites from these contigs in our alignment prior to SNP calling (Table 2). Using ANGSD, we identified 39,105 high‐quality SNPs with at least a 95% probability of being variable from 3,206 loci, for a matrix completeness of 62.8% (or 37.2% missing data across all individuals). Per individual, the proportion of missing sites ranged from 5.6% to 90.6%, with a significantly higher mean percentage missing data for historic samples (54.5%) than modern samples (28.3%) (*t = *6.727, *df *= 14.594, *p *> .001). The total number of SNPs in our data matrix decreased linearly after the first 10 individuals when we increased the minimum number of individuals successfully genotyped to retain each SNP (Figure 4).

### 3.4 Population genetic clustering and discriminant analysis of principal components

**Figure 5:**
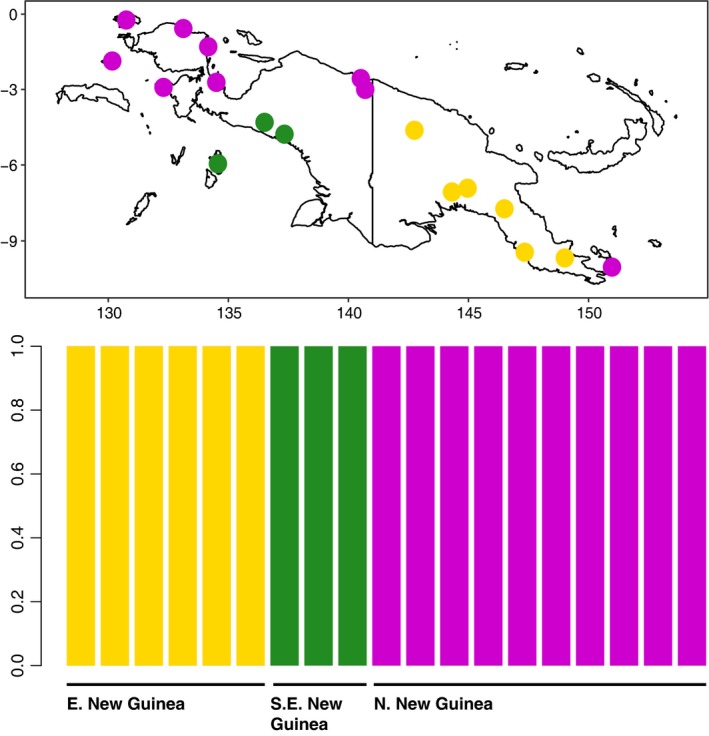
Individual membership probabilities for *K* = 3 ancestral populations inferred from analyzing 1,690 SNPs (100% complete data matrix) using discriminant analysis of principal components and retaining six principal component axes with two discriminant axes. Individual sampling locations are color coded accordingly

Discriminant analysis of principal components analysis of our 100% complete data matrix revealed a linear pattern of increase in the total amount of genetic variation explained when retaining additional PCs. Replicate attempts to optimize *a*‐score values alternatively suggested retaining either five or six PCs to maximize discrimination ability without overfitting the model. Similarly, BIC scores from DAPC decreased in an approximately linear fashion as more clusters were added and did not indicate a clear shift to a slower rate of BIC change. Therefore, we repeated our analysis for values of *K* from 1 to 8, which revealed patterns of increasingly fine, geographically coherent structure from *K* = 1 to *K *=* *5 (Figure 5). At *K* = 2, DAPC separated individuals from mainland Papua New Guinea (PNG) from individuals in western New Guinea and Normanby Island in PNG, which also reflected the break between modern and historic samples. At *K* = 3, DAPC identified an additional cluster from West Papua that included individuals from the southwest New Guinea Coast and the Aru Islands. At *K* = 4, DAPC isolated an individual from the northern slope of the Arfak Mountains in Western New Guinea, which, collected in 1877, was also the oldest sample included in our study. A fifth cluster distinguished the single individual from Normanby Island, PNG. At *K* = 6 and greater, DAPC began subdividing individuals into additional clusters without a shared geographic basis. Our Mantel test did not find statistically significant correlation between geographic and genetic distance (*p* = .251). Linear regression analyses showed significant correlations between all variables and PC1 but no other PC and sample variable pairs (Table 3).

**Table 3:** *P* values for simple linear regression between PC axes and sample variables

	Specificity	Sensitivity	Fold enrichment	Age	Initial concentration
PC1	<.001	<.001	<.001	<.001	<.001
PC2	.076	.534	.076	.242	.253
PC3	.437	.335	.437	.466	.552

## 4 DISCUSSION

### 4.1 Hybridization‐RAD is an effective tool for sampling thousands of orthologous SNPs from historic museum specimens

In their description of hyRAD, Suchan et al. (2016) suggest that it allows “sequencing of orthologous loci even from highly degraded DNA samples” and can be used to retrieve sequence data using museum samples up to 100 years old.

Our hybridization capture experiments in an independent laboratory with an independent study organism (*S. torotoro*) largely support this conclusion. Assessed by the total number assembled contigs, orthologous loci, SNPs collected, and number of SNPs recovered across multiple individuals (Table 1; Figure 4), our modified version of Suchan et al.'s hyRAD protocol generated sufficient quantities of genome‐wide data for a wide range of phylogenetic and population genetic questions. In particular, we note that our application of standard library preparation methods for both modern and historic libraries (as opposed to protocols optimized for degraded DNA, as in Suchan et al., 2016) does not appear to have negatively affected data recovery rates. We believe this reflects the relative robustness of the approach to different taxa, laboratory conditions, specimen preparation conditions, and bioinformatics pipelines. Even with stringent filtering for quality and postmortem damage, our 100% complete data matrix of 1,690 SNPs is similar to the number of orthologous SNPs collected in similar studies of museum genomics that used UCE capture (McCormack et al., 2016), exon sequence capture (Bi et al., 2013), or even ddRAD methods with fresh tissues (Shultz, Baker, Hill, Nolan, & Edwards, 2016), with the caveat that differences in starting material, evolutionary timescale, and experimental design preclude direct comparisons across studies. For phylogenetic or population genetic analyses methods that correct for nonrandom patterns of missing data, our full matrix of 39,105 SNPs potentially offers significant power to resolve rapid, recent divergences, detect fine scale patterns of population structure, infer historical effective population sizes with high accuracy, and reveal histories of drift, selection, and migration (Toews et al., 2016).

Suchan et al. (2016) included historical museum specimens up to 58 years old in their validation experiment and up to 100 years old in their pilot study. We successfully captured 55 on‐target loci, including four contigs exceeding 1 kb in length, from a specimen collected in 1877, and as many as 508 loci from specimens collected from 1896 to 1934 (Table 1). Moreover, and contrary to similar analyses by Suchan et al. (2016) and McCormack et al. (2016), we found no significant linear relationship between age and assembly or sequencing success metrics across historical samples. While this is possibly an artifact of small sample size relative to both previous studies, it may also suggest a relatively shallow trend of degradation during a period when many bird specimens in natural history museums were originally collected. This is an encouraging result for researchers looking to make use of these specimens as a genomic resource.

### 4.2 Variation in sequencing and assembly performance across sample types reduces efficiency

Our small sample size prevents us from making broad conclusions about factors affecting variation in enrichment, sequencing, and assembly performance. However, preliminary statistical analyses revealed variation between modern and ancient DNA libraries across numerous metrics. Modern DNA samples had significantly higher specificity (% mapped cleaned reads), sensitivity (% of probe sequence with at least 1× coverage), and fold enrichment (the fold increase in % mapped reads over baseline random expectations) (Figure 2). While initially intuitive, our findings contrast with the results of Bi et al. (2013) and Suchan et al. (2016), who found improved capture efficiency with historic samples, potentially related to smaller fragment size. We encourage future studies to explore how variation in hybridization capture protocols affects relative performance of different sample DNA sources.

Encouragingly, there were no significant differences between the two sample populations for the overall percentage of duplicate reads. However, we wish to highlight the high percentage of duplicate reads present in all samples (50.9%–91.1%). This may be due to combination of the relatively high number of amplification cycles used to amplify libraries with low input DNA prior to pooling. While high duplicate read percentages have also been reported in RADseq studies with fresh tissue (Andrews et al., 2016), this inefficiency is important to consider when working with valuable, low‐quality historical samples, as increased sequencing effort may be required to generate sufficient read depth for variant detection and accurate assembly of contiguous sequences.

Lastly, following assembly, the total number of captured on‐target loci was higher among modern samples, likely reflecting higher copy number and limited degradation of DNA from fresh tissues. In contrast, mean contig length did not vary significantly among samples, indicating similar assembly performance relative to the amount of high‐quality data for each sample type. While we believe this result will prove robust to different assembly methods, we encourage future studies to explore their influence on resulting assemblies and downstream analyses.

### 4.3 Input DNA quantity predicts GC content, suggesting PCR bias

Although inferences are similarly limited by sample size, our regression analyses largely failed to reveal significant correlations between input DNA/sequencing variables and assembly performance except in two comparisons (Figure 2). First, an increase in the number of filtered reads was positively correlated with the total number of assembled on‐target contigs, which matches a standard expectation of increased recovery with greater sampling of a genomic library with an uneven distribution of fragments, suggested for our libraries by the high levels of duplication (Table 1). Second, the initial quantity of input DNA in a sample was negatively correlated with %GC content in resulting assemblies, for example, the two samples in our study that with the lowest input DNA quantity also the highest percentage of GC content across all assembled contigs (Table 1). This finding may reflect biased PCR enrichment of GC‐rich exogenous microbial contamination in samples with low initial input DNA quantity (Dabney & Meyer, 2012) and explain the significantly higher GC content of historic samples overall. While our SNP calling pipeline and data filtering removed sites potentially originating from nonvertebrate sequences (although see further discussion below), we nonetheless recommend researchers interested in applying hyRAD to historical specimens heed the recommendations of recent empirical studies (Gamba et al., 2016) to select an extraction protocol suited to degraded DNA and maximize input tissue quantity whenever possible.

### 4.4 Geographic and/or taxonomic autocorrelation with input DNA type is potentially problematic with hyRAD studies

We implemented rigorous and conservative laboratory and bioinformatic protocols to reduce the influence of exogenous DNA contamination and postmortem DNA degradation, the results of which we summarize as a reference for future studies in Table 2. Despite these precautions, repeated DAPC with different parameters failed to change a basic pattern where all modern DNA samples (*n* = 6) clustered until the chosen *K* value of ancestral populations was seven or more (Figure 5). Unfortunately, as the close geographic proximity of modern DNA samples would also lead to this pattern, we could not easily determine from our sampling scheme if this pattern reflected biological reality or whether factors correlated with sample type, such as undetected microbial contamination, DNA degradation, and/or library amplification artifacts, were affecting population genetic inference. We first attempted to disentangle its potential drivers during PCA and DAPC analyses by examining histograms of %GC content per read for anomalous distributions, but failed to detect a significant second peak indicative of contamination with exogenous GC‐rich microbes. We next performed linear regressions with specificity, sensitivity, fold enrichment, specimen age, and input DNA quantity as predictors for each of our first three retained PCs. From these regression analyses, we found significant correlations of all variables with PC1, but no other PCs (Table 3). While these results are consistent with the possibility that biased sequencing performance affected population genetic inference, the exact mechanism responsible remains unclear. To avoid artifacts related to the separate treatment of different sample types, we suggest randomizing individuals from both modern and historic DNA sources throughout library preparation, hybridization capture, and sequencing. Additionally, we suggest researchers intending to use hyRAD in studies with both modern and historic tissue attempt to avoid geographic and taxonomic autocorrelation wherever possible and include a control in their sampling scheme to indicate potential DNA input quality problems.

### 4.5 The phylogeography of *S*. *torotoro* reflects biogeographic barriers in New Guinea

Our DAPC results are consistent with previous studies of lowland avian phylogeography in New Guinea, and we interpret them as independent confirmation the ability of hyRAD to reveal biologically meaningful patterns. *K*‐means clusters recovered for three ancestral populations reflect broad trends in codistributed taxa and expected patterns of genetic variation given geographic barriers and the geologic history of New Guinea (Figure 5) (Deiner et al., 2011; Dumbacher & Fleischer, 2001). Although IBD across all samples was not significant (see Section “3” for details), the initial division of samples into mainland eastern and western clusters is consistent with both the primary latitudinal axis of the island and the barriers to gene flow in lowland forest taxa presented by the Bewani Mountains and Trans‐Fly savannah region (Deiner et al., 2011; Mack & Dumbacher, 2007). Inclusion of Normanby Island subspecies *S. t. ochracea* in the western New Guinea cluster is consistent with previous studies that have reported genetic similarity between other taxa in far eastern and far western New Guinea, such as birds of the genus *Pitohui* (Dumbacher & Fleischer, 2001). Samples from southwest New Guinea clustered with those from the Aru Islands, suggesting shared ancestry among these currently allopatric populations. This is potentially explained by both the linkage of these landmasses during the Pleistocene via the Sahul Shelf (Voris, 2000) and the subsequent emergence of previously identified barriers to avian gene flow to the north, east, and west in the form of the Central Ranges, the Trans‐Fly Savannah, and Aetna Bay, respectively (Dumbacher & Fleischer, 2001; Deiner et al., 2011;.) Our analysis reveals broad similarities between the phylogeography of *S. torotoro* and the codistributed lowland bird species *Colluricincla megarhyncha* (Deiner et al., 2011), albeit with lower resolution due to the inherent limitations of our sampling. We believe that future studies of resident lowland forest species with similar ranges that use hyRAD or other means of capturing nuclear DNA markers will continue to aid in building a cohesive picture of the comparative phylogeography of this biodiverse region.

## CONFLICT OF INTEREST

None declared.

## AUTHOR CONTRIBUTIONS

EBL and JPD designed the research; AS and ZH developed the wet‐lab protocol; EBL and AS performed molecular work; EBL and ZH developed the bioinformatics pipeline and analyzed data; EBL wrote the manuscript with editorial contributions from all authors.

## References

[ece33065-bib-0001] Andrews, K. R. , Good, J. M. , Miller, M. R. , Luikart, G. , & Hohenlohe, P. A. (2016). Harnessing the power of RADseq for ecological and evolutionary genomics. Nature Reviews Genetics, 17(2), 81–92. 10.1038/nrg.2015.28 PMC4823021 26729255

[ece33065-bib-0002] Axelsson, E. , Willerslev, E. , Gilbert, M. T. P. , & Nielsen, R. (2008). The effect of ancient DNA damage on inferences of demographic histories. Molecular Biology and Evolution, 25, 2181–2187. 18653730 10.1093/molbev/msn163

[ece33065-bib-0003] Baird, N. A. , Etter, P. D. , Atwood, T. S. , et al. (2008). Rapid SNP discovery and genetic mapping using sequenced RAD markers. PLoS One, 3, e3376. 18852878 10.1371/journal.pone.0003376 PMC2557064

[ece33065-bib-0004] Bankevich, A. , Nurk, S. , Antipov, D. , et al. (2012). SPAdes: A new genome assembly algorithm and its applications to single‐cell sequencing. Journal of Computational Biology, 19, 455–477. 22506599 10.1089/cmb.2012.0021 PMC3342519

[ece33065-bib-0005] Besnard, G. , Bertrand, J. A. M. , Delahaie, B. , et al. (2015). Valuing museum specimens: High‐throughput DNA sequencing on historical collections of New Guinea crowned pigeons (*Goura*). Biological Journal of the Linnean Society, 117, 71–82.

[ece33065-bib-0006] Bi, K. , Linderoth, T. , Vanderpool, D. , et al. (2013). Unlocking the vault: Next‐generation museum population genomics. Molecular Ecology, 22, 6018–6032. 24118668 10.1111/mec.12516 PMC4134471

[ece33065-bib-0007] Bolger, A. M. , Lohse, M. , & Usadel, B. (2014). Trimmomatic: A flexible trimmer for Illumina sequence data. Bioinformatics, 30, 2114–2120. 24695404 10.1093/bioinformatics/btu170 PMC4103590

[ece33065-bib-0008] Boratyn, G. M. , Camacho, C. , Cooper, P. S. , et al. (2013). BLAST: A more efficient report with usability improvements. Nucleic Acids Research, 41, W29–W33. 23609542 10.1093/nar/gkt282 PMC3692093

[ece33065-bib-0009] Briggs, A. W. , Stenzel, U. , Johnson, P. L. F. , et al. (2007). Patterns of damage in genomic DNA sequences from a Neandertal. Proceedings of the National Academy of Sciences of the United States of America, 104, 14616–14621. 17715061 10.1073/pnas.0704665104 PMC1976210

[ece33065-bib-0010] Burbano, H. A. , Hodges, E. , Green, R. E. , et al. (2010). Targeted investigation of the Neandertal genome by array‐based sequence capture. Science, 328, 723–725. 20448179 10.1126/science.1188046 PMC3140021

[ece33065-bib-0011] Cooper, A. , Mourer‐Chauviré, C. , Chambers, G. K. , et al. (1992). Independent origins of New Zealand moas and kiwis. Proceedings of the National Academy of Sciences of the United States of America, 89, 8741–8744. 1528888 10.1073/pnas.89.18.8741 PMC49996

[ece33065-bib-0012] Dabney, J. , & Meyer, M. (2012). Length and GC‐biases during sequencing library amplification: A comparison of various polymerase‐buffer systems with ancient and modern DNA sequencing libraries. BioTechniques, 52, 87–94. 22313406 10.2144/000113809

[ece33065-bib-0013] Deiner, K. , Lemmon, A. R. , Mack, A. L. , Fleischer, R. C. , & Dumbacher, J. P. (2011). A passerine bird's evolution corroborates the geologic history of the Island of New Guinea. PLoS One, 6, e19479. 21573115 10.1371/journal.pone.0019479 PMC3089620

[ece33065-bib-0014] Dumbacher, J. P. , & Fleischer, R. C. (2001). Phylogenetic evidence for colour pattern convergence in toxic pitohuis: Mullerian mimicry in birds? Proceedings of the Royal Society B: Biological Sciences, 268, 1971–1976. 11571042 10.1098/rspb.2001.1717 PMC1088837

[ece33065-bib-0333] Eaton, D. A. (2014). PyRAD: Assembly of de novo RADseq loci for phylogenetic analyses. Bioinformatics, btu121. 10.1093/bioinformatics/btu121 24603985

[ece33065-bib-0016] Edgar, R. C. (2004). MUSCLE: Multiple sequence alignment with high accuracy and high throughput. Nucleic Acids Research, 32, 1792–1797. 15034147 10.1093/nar/gkh340 PMC390337

[ece33065-bib-0017] Faircloth, B. C. , McCormack, J. E. , Crawford, N. G. , et al. (2012). Ultraconserved elements anchor thousands of genetic markers spanning multiple evolutionary timescales. Systematic Biology, 61, 717–726. 22232343 10.1093/sysbio/sys004

[ece33065-bib-0018] Fleischer, R. C. , Kirchman, J. J. , Dumbacher, J. P. , et al. (2006). Mid‐Pleistocene divergence of Cuban and North American ivory‐billed woodpeckers. Biology Letters, 2, 466–469. 17148432 10.1098/rsbl.2006.0490 PMC1686174

[ece33065-bib-0020] Fu, L. , Niu, B. , Zhu, Z. , Wu, S. , & Li, W. (2012). CD‐HIT: Accelerated for clustering the next‐generation sequencing data. Bioinformatics, 28, 3150–3152. 23060610 10.1093/bioinformatics/bts565 PMC3516142

[ece33065-bib-0021] Gamba, C. , Hanghøj, K. , Gaunitz, C. , et al. (2016). Comparing the performance of three ancient DNA extraction methods for high‐throughput sequencing. Molecular Ecology Resources, 16, 459–469. 26401836 10.1111/1755-0998.12470

[ece33065-bib-0022] Gautier, M. , Gharbi, K. , Cezard, T. , et al. (2013). The effect of RAD allele dropout on the estimation of genetic variation within and between populations. Molecular Ecology, 22, 3165–3178. 23110526 10.1111/mec.12089

[ece33065-bib-0024] Guschanski, K. , Krause, J. , Sawyer, S. , et al. (2013). Next‐generation museomics disentangles one of the largest primate radiations. Systematic Biology, 62, 539–554. 23503595 10.1093/sysbio/syt018 PMC3676678

[ece33065-bib-0025] Habel, J. C. , Husemann, M. , Finger, A. , Danley, P. D. , & Zachos, F. E. (2014). The relevance of time series in molecular ecology and conservation biology. Biological Reviews of the Cambridge Philosophical Society, 89, 484–492. 24251767 10.1111/brv.12068

[ece33065-bib-0026] Hanna, Z. , & Sellas, A. (2016). hyRADccg wetlab protocol. https://github.com/calacademy-research/hyRADccg/

[ece33065-bib-0027] Henderson, J. B. , & Hanna, Z. R. (2016). GItaxidIsVert. Version 1.0.0 [Data set] . Zenodo. https://doi.org/10.5281/zenodo.163737

[ece33065-bib-0028] Hofreiter, M. , Jaenicke, V. , Serre, D. , von Haeseler, A. , & Pääbo, S. (2001). DNA sequences from multiple amplifications reveal artifacts induced by cytosine deamination in ancient DNA. Nucleic Acids Research, 29, 4793–4799. 11726688 10.1093/nar/29.23.4793 PMC96698

[ece33065-bib-0029] Hofreiter, M. , Paijmans, J. L. A. , Goodchild, H. , et al. (2015). The future of ancient DNA: Technical advances and conceptual shifts. BioEssays, 37, 284–293. 25413709 10.1002/bies.201400160

[ece33065-bib-0030] Jombart, T. (2008). adegenet: A R package for the multivariate analysis of genetic markers. Bioinformatics, 24, 1403–1405. 18397895 10.1093/bioinformatics/btn129

[ece33065-bib-0031] Jones, M. R. , & Good, J. M. (2016). Targeted capture in evolutionary and ecological genomics. Molecular Ecology, 25, 185–202. 26137993 10.1111/mec.13304 PMC4823023

[ece33065-bib-0032] Korneliussen, T. S. , Albrechtsen, A. , & Nielsen, R. (2014). ANGSD: Analysis of next generation sequencing data. BMC Bioinformatics, 15, 356. 25420514 10.1186/s12859-014-0356-4 PMC4248462

[ece33065-bib-0033] Langmead, B. (2010). Aligning short sequencing reads with Bowtie. Current Protocols in Bioinformatics, 32:11.7, 11.7.1–11.7.14. 10.1002/0471250953.bi1107s32 PMC3010897 21154709

[ece33065-bib-0034] Li, H. , Handsaker, B. , Wysoker, A. , et al. (2009). The sequence alignment/map format and SAMtools. Bioinformatics, 25, 2078–2079. 19505943 10.1093/bioinformatics/btp352 PMC2723002

[ece33065-bib-0035] Linck, E. , Schaack, S. , & Dumbacher, J. P. (2016). Genetic differentiation within a widespread “supertramp” taxon: Molecular phylogenetics of the Louisiade White‐eye (*Zosterops griseotinctus*). Molecular Phylogenetics and Evolution, 94, 113–121. 26327326 10.1016/j.ympev.2015.08.018

[ece33065-bib-0036] Liu, B. , Yuan, J. , Yiu, S.‐M. , et al. (2012). COPE: An accurate k‐mer‐based pair‐end reads connection tool to facilitate genome assembly. Bioinformatics, 28, 2870–2874. 23044551 10.1093/bioinformatics/bts563

[ece33065-bib-0015] Mack, A. , & Dumbacher, J. (2007). Birds of Papua. The Ecology of Papua Part 1 (pp. 654–688). Oxford, UK: Oxford University Press.

[ece33065-bib-0037] Magoc, T. , & Salzberg, S. L. (2011). FLASH: Fast length adjustment of short reads to improve genome assemblies. Bioinformatics, 27, 2957–2963. 21903629 10.1093/bioinformatics/btr507 PMC3198573

[ece33065-bib-0038] Martin, M. (2011). Cutadapt removes adapter sequences from high‐throughput sequencing reads. EMBnet.journal, 17, 10.

[ece33065-bib-0039] McCormack, J. E. , Faircloth, B. C. , Crawford, N. G. , et al. (2012). Ultraconserved elements are novel phylogenomic markers that resolve placental mammal phylogeny when combined with species‐tree analysis. Genome Research, 22, 746–754. 22207614 10.1101/gr.125864.111 PMC3317156

[ece33065-bib-0040] McCormack, J. E. , Tsai, W. L. E. , & Faircloth, B. C. (2016). Sequence capture of ultraconserved elements from bird museum specimens. Molecular Ecology Resources, 16, 1189–1203. 26391430 10.1111/1755-0998.12466

[ece33065-bib-0041] Narasimhan, V. , Danecek, P. , Scally, A. , et al. (2016). BCFtools/RoH: A hidden Markov model approach for detecting autozygosity from next‐generation sequencing data. Bioinformatics, 32, 1749–1751. 26826718 10.1093/bioinformatics/btw044 PMC4892413

[ece33065-bib-0042] Payne, R. D. , & Sorenson, M. D. (2002). Museum collections as sources of genetic data. Bonner zoologische Beitrage: Herausgeber: Zoologisches Forschungsinstitut und Museum Alexander Koenig, Bonn, 51, 97–104.

[ece33065-bib-0043] Peterson, B. K. , Weber, J. N. , Kay, E. H. , Fisher, H. S. , & Hoekstra, H. E. (2012). Double digest RADseq: An inexpensive method for *de novo* SNP discovery and genotyping in model and non‐model species. PLoS One, 7, e37135. 22675423 10.1371/journal.pone.0037135 PMC3365034

[ece33065-bib-0044] Poinar, H. N. , Schwarz, C. , Qi, J. , et al. (2006). Metagenomics to paleogenomics: Large‐scale sequencing of mammoth DNA. Science, 311, 392–394. 16368896 10.1126/science.1123360

[ece33065-bib-0045] Pratt, T. K. , & Beehler, B. M. (2014). Birds of New Guinea (2nd ed.). Princeton, NJ: Princeton University Press.

[ece33065-bib-0047] Quinlan, A. R. , & Hall, I. M. (2010). BEDTools: A flexible suite of utilities for comparing genomic features. Bioinformatics, 26, 841–842. 20110278 10.1093/bioinformatics/btq033 PMC2832824

[ece33065-bib-0048] R Core Team (2016). R: A language and environment for statistical computing. Vienna, Austria: R Foundation for Statistical Computing URL: https://www.R-project.org/

[ece33065-bib-0049] Rizzi, E. , Lari, M. , Gigli, E. , De Bellis, G. , & Caramelli, D. (2012). Ancient DNA studies: New perspectives on old samples. Genetics, Selection, Evolution: GSE, 44, 21. 10.1186/1297-9686-44-21 PMC3390907 22697611

[ece33065-bib-0050] Rognes, T. , Flouri, T. , Nichols, B. , Quince, C. , & Mahé, F. (2016). VSEARCH: A versatile open source tool for metagenomics. PeerJ, 4, e2584. 27781170 10.7717/peerj.2584 PMC5075697

[ece33065-bib-0051] Shultz, A. J. , Baker, A. J. , Hill, G. E. , Nolan, P. M. , & Edwards, S. V. (2016). SNPs across time and space: Population genomic signatures of founder events and epizootics in the House Finch (*Haemorhous mexicanus*). Ecology and Evolution, 6, 7475–7489. 10.1002/ece3.2444 PMC5513257 28725414

[ece33065-bib-0052] Soltis, P. S. , & Soltis, D. E. (1993). Ancient DNA: Prospects and limitations. New Zealand Journal of Botany, 31, 203–209.

[ece33065-bib-0053] Suchan, T. , Pitteloud, C. , Gerasimova, N. S. , et al. (2016). Hybridization capture using RAD probes (hyRAD), a new tool for performing genomic analyses on collection specimens. PLoS One, 11, e0151651. 26999359 10.1371/journal.pone.0151651 PMC4801390

[ece33065-bib-0054] Thioulouse, J. , & Dray, S. (2007). Interactive multivariate data analysis in R with the ade4 and ade4TkGUI packages. Journal of Statistical Software, 22(5), 1–14.

[ece33065-bib-0055] Toews, D. P. L. , Campagna, L. , Taylor, S. A. , et al. (2016). Genomic approaches to understanding population divergence and speciation in birds. The Auk, 133, 13–30.

[ece33065-bib-0056] Voris, H. K. (2000). Maps of Pleistocene sea levels in Southeast Asia: Shorelines, river systems and time durations. Journal of Biogeography, 27, 1153–1167.

[ece33065-bib-0057] Wandeler, P. , Hoeck, P. E. A. , & Keller, L. F. (2007). Back to the future: Museum specimens in population genetics. Trends in Ecology & Evolution, 22, 634–642. 17988758 10.1016/j.tree.2007.08.017

[ece33065-bib-0058] Weber, D. S. , Stewart, B. S. , Garza, J. C. , & Lehman, N. (2000). An empirical genetic assessment of the severity of the northern elephant seal population bottleneck. Current Biology, 10, 1287–1290. 11069110 10.1016/s0960-9822(00)00759-4

[ece33065-bib-0059] Wójcik, J. M. , Kawałko, A. , Marková, S. , Searle, J. B. , & Kotlík, P. (2010). Phylogeographic signatures of northward post‐glacial colonization from high‐latitude refugia: A case study of bank voles using museum specimens. Journal of Zoology, 281, 249–262.

